# Impact of oral nutrition supplementation on outcomes of esophageal cancer patients treated with chemotherapy: A retrospective cohort study with propensity score matching

**DOI:** 10.3389/fnut.2022.1004372

**Published:** 2022-11-23

**Authors:** Xuemei Li, Tingting Dai, Zhiyong Rao, Wen Hu

**Affiliations:** Department of Clinical Nutrition, West China Hospital, Sichuan University, Chengdu, China

**Keywords:** esophageal cancer, propensity score matching, pulmonary infection, myelosuppression, oral nutrition supplementation

## Abstract

**Objective:**

There is a lack of evidence regarding the outcomes of oral nutrition supplementation (ONS) in patients with esophageal cancer (EC) who received chemotherapy treatment. The aim of this study was to perform a retrospective cohort study by comparing an adequate ONS group with a control group.

**Materials and methods:**

The study was performed in the Oncology Department of West China Hospital of Sichuan University. Patients at nutritional risk were identified from March 2016 to June 2019, and divided into an ONS group and a control group. To control for potential confounding variables, the propensity score method with matching was carried out. The main outcomes were length of stay (LOS) and hospitalization cost. Secondary outcomes included the incidence of pulmonary infection and myelosuppression.

**Results:**

Out of 5,316 hospitalizations, a one-to-one matched sample was created (*N* = 229). The pathological tumor, node, metastasis (pTNM) stage of patients ranged from II to IV. A total of 69 patients received ONS, and 160 patients did not receive ONS. The incidence of myelosuppression in the ONS group and the control group was 4.3 vs. 17.4% (*P* = 0.014), respectively. However, ONS was associated with a 2 days increase in LOS, from 7 to 9 days (*P* < 0.000) and a hospitalization cost increase of $731, from $1134 to $1865 (*P* = 0.005). No statistical differences were observed in the incidence of pulmonary infection between the two groups. Further subgroup analysis based on body mass index (BMI) showed that at BMI ≤ 18.5 kg/m^2^, the incidence of myelosuppression in the ONS group was lower than that in the control group (3.0 vs. 20.8%, *P* = 0.022). At BMI > 18.5 kg/m^2^, no statistical differences were observed in the incidence of myelosuppression between the two groups.

**Conclusion:**

Although ONS increases hospitalization cost and LOS, it may be associated with reduced myelosuppression incidence, especially for patients with a BMI ≤ 18.5 kg/m^2^.

## Introduction

Esophageal cancer (EC) is one of the most common malignant gastrointestinal neoplasms (1). In 2020, the number of new EC cases reached 604,000 (1), of which 53.7% of cases were in China (2). EC is a significant health challenge because its disability adjusted life years (DALY) ranks fourth among all malignant tumors (3). No matter the progress of the disease or the side effects of radiotherapy, it may cause dysphagia in patients with EC resulting in malnutrition (4). Malnutrition will increase the mortality of EC patients (5, 6), thus, it is necessary to treat EC patients with nutrition therapy. In present studies, increased attention is paid to the effects of nutrition treatment on the nutritional status (7, 8), immune indicators (9) of EC patients. However, few reports are available on the evaluation of clinical outcomes, such as complications, length of stay (LOS), and hospitalization costs. Moreover, most current related studies are randomized controlled trials (RCT), and no data are available on the impact of oral nutrition supplementation (ONS). Therefore, the purpose of this study was to investigate the effect of ONS on the outcomes of EC patients who received chemotherapy in West China Hospital of Sichuan University using propensity score matching (PSM).

## Materials and methods

### Participants

This study was a retrospective observational study conducted in the oncology department of West China Hospital of Sichuan University. Recruitment was carried out among patients admitted consecutively to the department from March 2016 to June 2019.

Patients were considered eligible if they met the following criteria: (1) over the age of 18; (2) primary EC diagnosed by pathology; (3) treated with chemotherapy and prior chemotherapy was administered; (4) at nutritional risk (NRS2002 score ≥ 3). Nutritional screening is the standard practice in this hospital, only patients at nutritional risk could receive ONS.

Exclusion criteria were as follows: (1) LOS ≤ 5 days; (2) pregnant or breastfeeding women; (3) combined with a primary malignant tumor in other parts of the body; (4) patients who gave up treatment and left the hospital voluntarily.

### Study design

This retrospective cohort study aimed to investigate the effect of ONS on the clinical outcome of EC patients treated with chemotherapy using the PSM method. The patients’ ID, gender, age, height, weight, tumor stage, chemotherapy plan, ONS treatment plan, radical operation history, history of lung infection, and myelosuppression, admission date, discharge date, and hospitalization cost were collected. Patients provided written informed consent to participate in this study. The study was registered on the Chinese Clinical Trial Registry as ChiCTR1900025146. The study protocol was approved by the ethics committee of West China Hospital, Sichuan University (No. 2019-725). Moreover, the study was conducted according to the Declaration of Helsinki.

### Definitions

(1) Tumor stage: tumor, node, metastasis (TNM) stage standard, according to the 7th edition of American Joint Committee on Cancer (AJCC).

(2) Nutritional risk: refers to the risk of adverse clinical outcomes of patients caused by existing or potential nutrition-related factors, rather than by malnutrition. In this study, NRS2002 score ≥ 3 indicated nutritional risk.

(3) Oral nutrition supplementation: refers to the nutritional treatment method for patients to obtain the energy and nutrients needed by the body through food for special medical purposes (FSMP) for more than 5 days. The FSMP used in this study was hypercaloric (≥ 1.3 kcal/ml), protein-enriched (>4 g/100 ml), polymeric formula administered by bolus.

(4) Parenteral nutrition (PN): refers to the administration of glucose, fat emulsion, and amino acids through the parenteral route.

(5) Pulmonary infection: refers to inflammation of the terminal airways, alveoli and pulmonary interstitium, which can be caused by pathogenic microorganisms, physical and chemical factors, immune damage, allergies, and drugs. In this study, pulmonary infection refers to pneumonia due to bacterial or viral pathogens, or radiation pneumonia.

(6) Myelosuppression: refers to the 5th edition of the evaluation standard for common adverse events (CTCAE) issued by the National Cancer Research Institute of the United States, and meets any of the following requirements (10): (1) neutrophils < 1.5 × 10^9^/L; (2) platelets < 75 × 10^9^/L; (3) hemoglobin < 100 g/L; (4) total lymphocytes < 0.8 × 10^9^/L.

### Statistical analysis

A PSM cohort was designed to limit the effects of confounding factors when estimating treatment effects and side effects, given the non-randomized property of the study and the variety of factors that can influence the outcomes. Data from the two groups were inputted into the SPSS 22.0 system (Chicago, IL, USA). First, the propensity score was estimated by logistic regression. Seven variables were set as variables to be balanced: gender, age, body mass index (BMI), tumor stage, radical operation history, history of pulmonary infection and myelosuppression. A one-to-one matched analysis using nearest-neighbor matching was performed based on the estimated propensity score of each patient. A match was made when one patient in the ONS group had an estimated score within 0.2 SDs (caliper value) of another patient in the control group. Menu options were as follows: sampling without replacement, maximized execution performance, and randomized case order when drawing matches.

In this study, the continuous data did not follow a normal distribution, therefore, the data are expressed by frequencies and percentages for categorical variables and by the median (interquartile range) for continuous variables. Continuous variables were compared using the Mann-Whitney U test and categorical variables were compared using the Chi-square test or Fisher’s exact test. A difference of *P* < 0.05 between two groups was considered statistically significant.

## Results

### Patient characteristics

**FIGURE 1:**
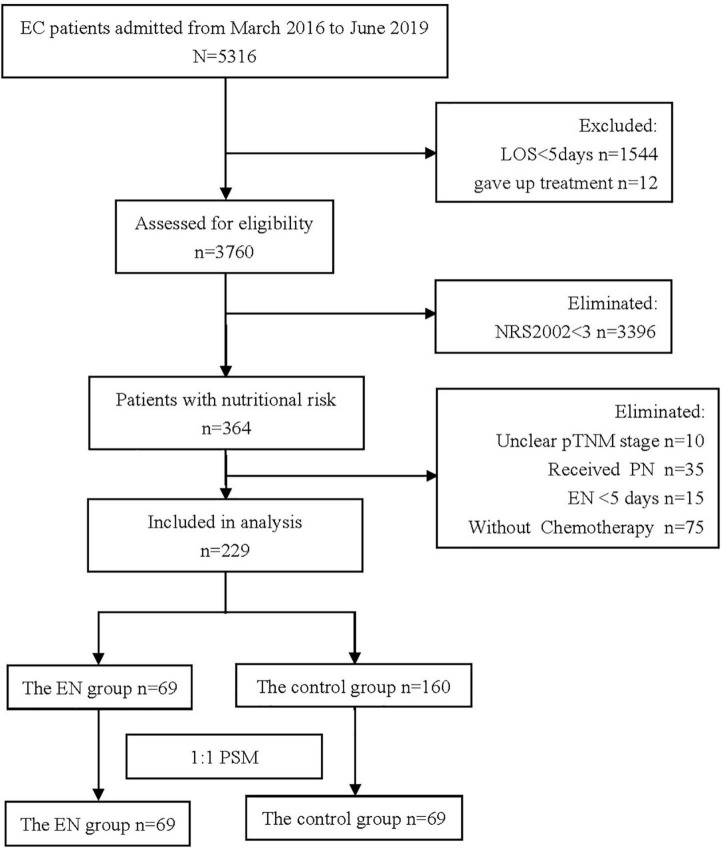
Flow chart of cohort (It shows the process of screening the patients treated or untreated with oral nutrition supplementation (ONS) in the initial and final propensity score matched sample).

A total of 5,316 patients were registered during the data collection period and 229 patients fulfilled the inclusion criteria (Figure 1). ONS was given to 69 patients, and 160 patients did not receive ONS. The baseline data of the two groups before and after PSM are shown in Table 1. Before PSM, the ONS group and the control group were poorly balanced for BMI (*P* = 0.049), pneumonia incidence at the time of admission (*P* = 0.011), and myelosuppression incidence at the time of admission (*P* = 0.036). The balance of other indicators was good. After PSM, there were no statistical differences in baseline characteristics between groups among the matched population, except for BMI (*P* = 0.007). Therefore, subgroup analysis was carried out for the BMI, and patients were divided into two subgroups: BMI > 18.5 kg/m^2^ and BMI ≤ 18.5 kg/m^2^. Baseline data and *P*-values of the patients’ propensity scores before and after matching in the two groups are shown in Table 2. The baseline indicators of patients in both groups were well-balanced.

**TABLE 1:** Characteristics of esophageal cancer (EC) patients before and after propensity score matching (PSM).

Item	Before PSM (*N* = 229)			After PSM (*N* = 138)		
	The EN group	The control group	*P-value*	The EN group	The control group	*P-value*
				
	*n* = 69 (Q1–Q3 or %)	*n* = 160 (Q1–Q3 or %)		*n* = 69 (Q1–Q3 or %)	*n* = 69 (Q1–Q3 or %)	
Male	60 (87.0)	146 (91.3)	0.321	60 (87.0)	66 (95.7)	0.07
Age (year)	62 (54–65)	61 (53–66)	0.628	62 (54–65.5)	60 (55–67)	0.871
BMI (kg/m^2^)	19.0 (17.9–21.5)	18.3 (17.3–20.0)	0.049	19.0 (17.9–21.5)	17.9 (17.3–19.25)	0.007
pTNM stage	–	–	0.103		–	0.065
II	6 (8.7)	30 (18.8)	–	6 (8.7)	16 (23.2)	–
III	43 (62.3)	80 (50.0)	–	43 (62.3)	35 (50.7)	–
IV	20 (29.0)	50 (31.3)	–	20 (29.0)	18 (26.1)	–
After radical resection	30 (43.5)	104 (65.0)	0.002	30 (43.5)	27 (39.1)	0.604
Pulmonary infection on admission	15 (21.7)	15 (9.4)	0.011	15 (21.7)	8 (11.6)	0.11
Myelosuppression on admission	3 (4.3)	22 (13.8)	0.036	4 (5.8)	10 (14.5)	0.091

**TABLE 2:** Characteristics of esophageal cancer (EC) patients with different body mass index (BMI).

Item	BMI ≤ 18.5 (*N* = 81)				BMI > 18.5 (*N* = 57)	
	The EN group *n* = 33 (Q1–Q3 or %)	The control group *n* = 48 (Q1–Q3 or %)	*P-value*	The EN group *n* = 36 (Q1–Q3 or %)	The control group *n* = 21 (Q1–Q3 or %)	*P-value*
Male	30 (90.9)	46 (95.8)	0.366	30 (83.3)	20 (95.2)	0.187
Age (year)	60 (53.5–64)	61 (55–66.75)	0.485	64 (54–66)	59 (54.5–70)	0.728
BMI (kg/m^2^)	17.9 (17.05–18.2)	17.5 (17.125–17.9)	0.427	21.5 (19.9–22.725)	20.8 (19.4–23.25)	0.608
pTNM stage	–	–	0.086	–	–	0.704
II	2 (6.1)	12 (25.0)	–	4 (11.1)	4 (19.0)	–
III	20 (60.6)	23 (47.9)	–	23 (63.9)	12 (57.1)	–
IV	11 (33.3)	13 (27.1)	–	9 (25.0)	5 (23.8)	–
After radical operation	14 (42.4)	19 (39.6)	0.789	16 (44.5)	8 (38.1)	0.640
Pulmonary infection on admission	3 (9.1)	5 (10.4)	0.844	12 (33.3)	3 (14.3)	0.115
Myelosuppression on admission	1 (3.0)	8 (16.7)	0.055	3 (8.3)	2 (9.5)	0.878

The incidence of myelosuppression in the ONS group and the control group was 4.3 vs. 17.4% (*P* = 0.014), respectively. However, ONS was associated with a 2 days increase in LOS, from 7 to 9 days (*P* < 0.000) and a hospitalization cost increase of $731, from $1134 to $1865 (*P* = 0.005), shown in Table 3. No statistical differences were observed in the incidence of pulmonary infection between the two groups. After statistical analysis of subgroups, it was found that when BMI ≤ 18.5 kg/m^2^, there was no statistical difference in the incidence of pulmonary infection and hospitalization cost between the two groups. The incidence of myelosuppression in the ONS group and the control group during hospitalization was 3.0 and 20.8%, respectively (*P* = 0.022). The LOS of patients in the ONS group and the control group was 9 and 7 days, respectively (*P* = 0.001). When BMI > 18.5 kg/m^2^, there was no statistical difference between the two groups in the incidence of pulmonary infection, myelosuppression, LOS and hospitalization cost, shown in Table 4.

**TABLE 3:** Clinical outcomes of esophageal cancer (EC) patients after propensity score matching (PSM) (*N* = 138).

Item	The EN group *n* = 69 (Q1–Q3 or %)	The control group *n* = 69 (Q1–Q3 or %)	*P-value*
Pulmonary infection	1 (1.4)	1 (1.4)	1.000
Myelosuppression	3 (4.3)	12 (17.4)	0.014
LOS (day)	9 (7–11)	7 (6–10)	< 0.001
Hospitalization cost (US dollars)	1865 (1319–2332)	1134 (687–1769)	0.005

**TABLE 4:** Clinical outcomes of esophageal cancer (EC) patients with different BMI after propensity score matching (PSM) (*N* = 138).

	BMI ≤ 18.5 kg/m^2^ (*N* = 81)			BMI > 18.5 kg/m^2^ (*N* = 57)		
Item	The EN group *n* = 33 (Q1–Q3 or %)	The control group *n* = 48 (Q1–Q3 or %)	*P-value*	The EN group *n* = 36 (Q1–Q3 or %)	The control group *n* = 21 (Q1–Q3 or %)	*P-value*
Pulmonary infection	0 (0)	1 (2.1)	1.000	1 (2.8)	0 (0)	1.000
Myelosuppression	1 (3.0)	10 (20.8)	0.022	2 (5.6)	2 (9.5)	0.620
LOS (day)	9 (7–11)	7 (6–10)	0.001	9 (7–11.75)	7 (6–8.5)	0.007
Hospitalization cost (US dollars)	1845 (1331–2343)	1085 (666–1735)	0.125	1876 (1317–2327)	1522 (776–2022)	0.053

## Discussion

Radiotherapy and chemotherapy are the main treatment methods for EC patients. Both therapies inevitably kill the immune cells, resulting in the inhibition of immune response, and various side effects, including leukopenia and thrombocytopenia (11). Myelosuppression not only reduces the quality of life of patients, but may also lead to the interruption of treatment and the reduction of the dose of anticancer drugs, which in turn reduces the effectiveness of chemotherapy treatment (12). In previous studies, it was shown that the 5 years overall survival rate of the myelosuppression group was significantly lower than that of the non-myelosuppression group (15.4 vs. 69.0%, *P* = 0.003). In addition, the incidence of preoperative chemotherapy interruption and chemotherapy drug dose reduction in the myelosuppression group was significantly higher than that in the non-myelosuppression group (*P* = 0.003) (13).

The hypothesis that EN is beneficial in reducing infectious complications is supported by evidence that shows that the induction of anabolism changes the immune reaction, thereby reducing inflammation (14). EN influences the ability of gut-associated lymphoid tissue to maintain mucosal immunity (15). The data in this study show that ONS may be associated with reduced myelosuppression incidence in hospitalized EC patients with nutritional risk (4.3 vs. 17.4%, *P* = s0.014). Although ONS increases hospitalization cost and LOS, we believe that the use of ONS is beneficial, because it may prevent myelosuppression. Furthermore, Cong and his colleagues (16) reported that the incidence of myelosuppression in the nutrition support team (NST) group and the control group was 20 and 48%, respectively (*P* = 0.037). However, in their study, patients with severe malnutrition (weight loss over 10%, albumin < 30 g/L or BMI < 18.5 kg/m^2^) were excluded. In another study, it was shown that, leukopenia and neutropenia (grade 3 or 4) were significantly less frequent in the EN group than the PN group (leukopenia: 17 vs. 41%, *P* = 0.011, neutropenia: 36 vs. 66%, *P* = 0.005), while total calories during chemotherapy were equal between groups (17). Furthermore, Han and his colleagues (18) showed that, compared with the total PN, the early EN reduced the postoperative length of hospital stay and hospital charges of Chinese EC patients who underwent esophagectomy.

It is worth mentioning that most studies related to EN focus on comparing the difference in curative effect between different nutrition treatment approaches (19), different formulas (20–25), and the starting time of nutrition treatment (26–29). Only few studies focused on the influence of ONS on the clinical outcome of EC inpatients. The Nutritional Risk Screening 2002 (NRS2002) tool is a nutritional assessment tool that is validated by evidence-based methodology (30). Patients with NRS2002 score ≥3 are at nutritional risk. The in-hospital mortality rate of patients with nutritional risk was 3.7 times higher than that of patients without nutritional risk (31), and NRS2002 could also predict the clinical outcome of EC patients. Cox regression analyses revealed that the TNM stage and NRS2002 score at baseline were independent risk factors for predicting the long-term outcome in patients with EC after radiochemotherapy (32). Therefore, NRS2002 was used in routine practice in the hospital to screen patients, to quickly identify patients who need nutrition treatment, and to make nutrition treatment more accurate (33). China has been facing a shortage of medical resources, and medical service providers are under great pressure (34). NRS2002 can avoid the huge waste of human and financial resources, which is worthy of national promotion. According to the requirements of China’s National Catalogue of Drugs for Basic Medical Insurance, Work Injury Insurance and Maternity Insurance in 2021, before the start of PN and EN treatment, patients need to be screened for nutritional risk, and only those with nutritional risk should be reimbursed. The proposal of this approach has greatly promoted the work of nutritional risk screening.

One of the strengths of our study is that our study is fully based on a real clinical scenario. Through PSM, the possible ethical problems caused by allocating patients into an ONS group and a control group were prevented, and data randomness and balance were further enhanced. At present, PSM has been used in relevant studies (35–37). To the best of our knowledge, this is the first study that has estimated the protective effect of ONS protecting myelosuppression in EC patients with nutritional risk using observational data by employing quasi-experimental statistical methods, such as PSM.

Our study has several limitations. First, it is a retrospective study and may have confounding factors and selection bias. For example, when collecting data in the early stage of this study, the timing of pulmonary infections was not considered, and our data showed that the incidence of pulmonary infections in the ONS group was higher than that in the control group. Later, we found that many patients already had a pulmonary infection before admission. After balancing the baseline of this factor, no statistical difference were observed in the incidence of pulmonary infections between groups. Therefore, other potential confounding factors may not be considered when balancing the baseline of the two groups of patients in this study. Second, several patients were excluded due to an unclear tumor stage, and because some samples were lost, because of the retrospective nature of this study. Third, in this study, the ONS group did not receive PN treatment, and all patients were treated with ONS, with an average energy supply of 548 kcal and an average treatment duration of 7.5 days. Patients with EC often have dysphagia and difficulty eating. However, the energy supply of patients in the ONS group may be insufficient. Moreover, the time of follow-up was limited, and long-term indicators, such as the survival rate of patients in the two groups were not observed. Finally, we regret that we have not made a more comprehensive analysis of unplanned healthcare resources to estimate potential savings in the use of medical resources (i.e., hospitalizations prevented, outpatient visits, emergency visits, household visits, or ambulance uses).

In conclusion, our data indicate that although ONS increases hospitalization cost and LOS, it may be associated with reduced myelosuppression incidence. Therefore, ONS could have a positive clinical value in the prevention of myelosuppression in EC patients with nutritional risk.

## Data availability statement

The raw data supporting the conclusions of this article will be made available by the authors, without undue reservation.

## Ethics statement

The studies involving human participants were reviewed and approved by the Ethics Committee of West China Hospital, Sichuan University. The patients/participants provided their written informed consent to participate in this study.

## Author contributions

XL: conceptualization. WH: validation. XL and TD: writing—original draft. ZR: writing—review and editing. All authors contributed to the article and approved the submitted version.
